# Assessment of the Presence of Pharmaceutical Compounds in Seawater Samples from Coastal Area of Gran Canaria Island (Spain)

**DOI:** 10.3390/antibiotics2020274

**Published:** 2013-05-30

**Authors:** Cristina Afonso-Olivares, Mª Esther Torres-Padrón, Zoraida Sosa-Ferrera, José Juan Santana-Rodríguez

**Affiliations:** Departamento de Química, Universidad de Las Palmas de Gran Canaria, Las Palmas de Gran Canaria 35017, Spain; E-Mails: cristina.afonso102@alu.ulpgc.es (C.A.-O.); mtorres@dqui.ulpgc.es (M.E.T.-P.); zsosa@dqui.ulpgc.es (Z.S.-F.)

**Keywords:** pharmaceutical compounds, SPE, LC-MS/MS, seawater, outfall

## Abstract

This study presents the evaluation of seven pharmaceutical compounds belonging to different commonly used therapeutic classes in seawater samples from coastal areas of Gran Canaria Island. The target compounds include atenolol (antihypertensive), acetaminophen (analgesic), norfloxacin and ciprofloxacin (antibiotics), carbamazepine (antiepileptic) and ketoprofen and diclofenac (anti-inflammatory). Solid phase extraction (SPE) was used for the extraction and preconcentration of the samples, and liquid chromatography tandem mass spectrometry (LC-MS/MS) was used for the determination of the compounds. Under optimal conditions, the recoveries obtained were in the range of 78.3% to 98.2%, and the relative standard deviations were less than 11.8%. The detection and quantification limits of the method were in the ranges of 0.1–2.8 and 0.3–9.3 ng·L^−1^, respectively. The developed method was applied to evaluate the presence of these pharmaceutical compounds in seawater from four outfalls in Gran Canaria Island (Spain) during one year. Ciprofloxacin and norfloxacin were found in a large number of samples in a concentration range of 9.0–3551.7 ng·L^−1^. Low levels of diclofenac, acetaminophen and ketoprofen were found sporadically.

## 1. Introduction

The rapid development of human civilisation has led to numerous environmental problems that affect water resources and has resulted in growing water shortages in various regions [1]. Exposure to hazardous chemicals including biocides, pesticides, and endocrine disrupters represents a threat that should be subject to assessment measures and the reduction and control of irrigation as specified by legislation [2]. Among the various laws relating to water is the EU Water Framework Directive 2000/60/EC (WFD) [3]. The WFD is a norm that provides a framework for community action in the area of water policy for the protection and management of inland waters, transitional waters, coastal waters and groundwater.

Traditionally, scientists have studied the presence of chemical pollutants that are regulated by law in the environment. However, more sensitive analytical methods have been developed and have made possible the detection of other hazardous pollutants, referred to as emerging contaminants [4,5], including pharmaceutical compounds [6,7]. Emerging pollutants are anthropogenic in origin; therefore, there are no natural background levels in the environment. The presence of pharmaceuticals in water due to agricultural, industrial and urban activities has been detected, with major sites of pollution being in wastewater treatment plants (WWTPs), sediments of rivers and coastal waters [8,9]. The presence of pharmaceutical compounds has been reported by several authors [10,11,12].

Currently, the preferred method for the determination of pharmaceuticals is high-resolution liquid chromatography with mass spectrometry detection (LC-MS) [13,14]. Despite the high sensitivity that can be obtained with this system, it is necessary to apply an extraction technique for sample preparation for analysis due to the low concentrations (ng·L^−1^) of these compounds in water samples. Solid phase extraction (SPE) is a commonly used sample pre-treatment technique. This methodology represents an attractive alternative to conventional liquid-liquid extraction techniques because it reduces the volume of organic solvent consumed, reduces the total analysis time and allows for automation [15,16].

In this work, we developed an analytical method based on solid-phase extraction (SPE) coupled to liquid chromatography tandem mass spectrometry (LC-MS/MS) for the determination of pharmaceutical compounds from different therapeutic classes in seawater samples. Table 1 shows the compounds studied and their physicochemical characteristics, which influence their behavior in the environment. The method was applied to evaluate the presence of these pharmaceutical compounds in seawater near outfalls of wastewater in Gran Canaria Island (Spain) between January 2011 and January 2012.

## 2. Experimental

### 2.1. Chemicals and Reagents

The pharmaceutical compounds selected in this study (atenolol, acetaminophen, norfloxacin, ciprofloxacin, carbamazepine, ketoprofen and diclofenac) were purchased from Sigma-Aldrich (Madrid, Spain). Their stock solutions (1 g·L^−1^) were prepared by dissolving appropriate amounts of pharmaceutical standards in methanol (HPLC gradient-grade PAI-ACS) from Panreac Química (Barcelona, Spain) and the solutions were then stored in glass stoppered bottles at 4 °C prior to use.

**Table 1:** List of pharmaceutical compounds, chemical structure and pKa values.

Compound	Use	Structure	pK_a_ [17,18]	K_ow_ *
**Atenolol**	Antihypertensive	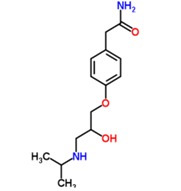	9.2	0.16
**Acetaminophen**	Analgesic	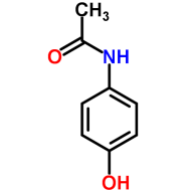	9.9	0.46
**Norfloxacin**	Antibiotic	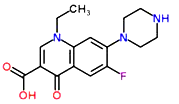	6.4	0.46
**Ciprofloxacin**	Antibiotic	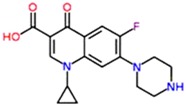	6.4	0.28
**Carbamazepine**	Antiepileptic	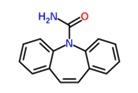	13.9	2.45
**Ketoprofen**	Anti-inflammatory	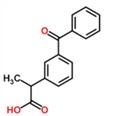	4.4	4.23
**Diclofenac**	Anti-inflammatory	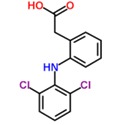	4.2	4.51

* Extracted from Hazardous Substances Data Bank.

LC-MS quality methanol and water were used to prepare the mobile phase for LC-MS/MS. All solvents and the ammonium formate and formic acid that were used to adjust the pH of the mobile phase were obtained from Panreac Química (Barcelona, Spain).

Ultra high purity water was obtained from a Milli-Q (Millipore, Bedford, MA, USA) water purification system and was used for conditioning the process of solid-phase extraction and for preparing aqueous standard solutions.

### 2.2. Sample Collection

The seawater samples for the determination of pharmaceutical compounds were collected in coastal areas near four outfalls from different urban wastewater treatment plants located on the island of Gran Canaria (Spain) during one year: Las Palmas de Gran Canaria (15.39351° W, 28.09292° N), Jinámar (15.38990° W, 28.03802° N), Punta Salina (15.40751° O, 27.84004° N), Los Cochinos (15.54993° W, 27.76447° N). 

The samples were collected at a depth of 1 m at the coordinates described and were stored in 1 L amber glass bottles that were pre-rinsed with methanol, deionised water and a real seawater sample for each sampling point. The samples were filtered through 0.65 µm membrane filters (Millipore, Cork, Ireland) and stored in the dark in a refrigerator. All samples were analysed within 48 h of collection.

### 2.3. Chromatographic Conditions

The chromatographic system from Varian (Varian Inc., Madrid, Spain) used for analysis of the selected pharmaceuticals compounds consisted of a 212-LC Binary Gradient LC-MS Chromatography Pump fitted with a Prostar 410 HPLC Autosampler and a 320-MS LC-MS/MS system (triple quadrupole) equipped with an electrospray ionisation (ESI) interface. The system and the data management were controlled and treated by MS Varian LC-MS Workstation Version 6.9 SP1 software.

The multiple reaction monitoring (MRM) parameters were optimised for subsequent quantitative analysis. This procedure was conducted using a 1 mL syringe (Hamilton Company, Reno, NV, USA) and a continuous flow rate of 20 µL·min^−1^. Each standard or mixture was prepared at a concentration of 10 mg·L^−1^ in methanol. Each solution was taken up with a Hamilton syringe at a volume of 0.1 mL and the remaining 0.9 mL of syringe volume was filled with mobile phase.

Ionisation in the ESI source was achieved using nitrogen as the nebuliser and drying gas. The housing and desolvation temperatures were set to 60 °C and 250 °C, respectively, for the optimisation of the syringe pump injections for MS/MS. The drying and nebulising gas pressures were fixed at 30 psi and 65 psi, respectively. The capillary voltage was set to 4.5 kV in positive mode (ESI+) and −3 kV in negative mode (ESI−). The shield voltage was maintained at −600/600 V (ESI+/ESI−) and the cone voltage was optimised for each individual compound. Collision-induced dissociation (CID) was conducted with argon as the collision gas at a fixed pressure of 1.94 psi.

The analytical column used for the separation was a 2.0 mm × 50 mm, 2.4 µm particle size Pursuit UPS column (Agilent Technologies). The mobile phase consisted of water (containing 0.2% formic acid and 5 mM ammonium formate, pH 2.6) and methanol. The program began at (90:10) (v/v) for 8 min, then 80:20 (v/v) (water/methanol) for 2 min, followed by an increase to 50% (v/v) of methanol over 5 min, a further increase to 100% (v/v) of methanol over 10 min, and lastly, a return to the initial conditions over 3 min. The system was then allowed to equilibrate for 2 min. The injection volume was 10 µL, and the flow rate was 200 µL·min^−1^. Calibration of the pharmaceutical compounds was performed in the range of 0.5–600 µg·L^−1^.

**Figure 1:**
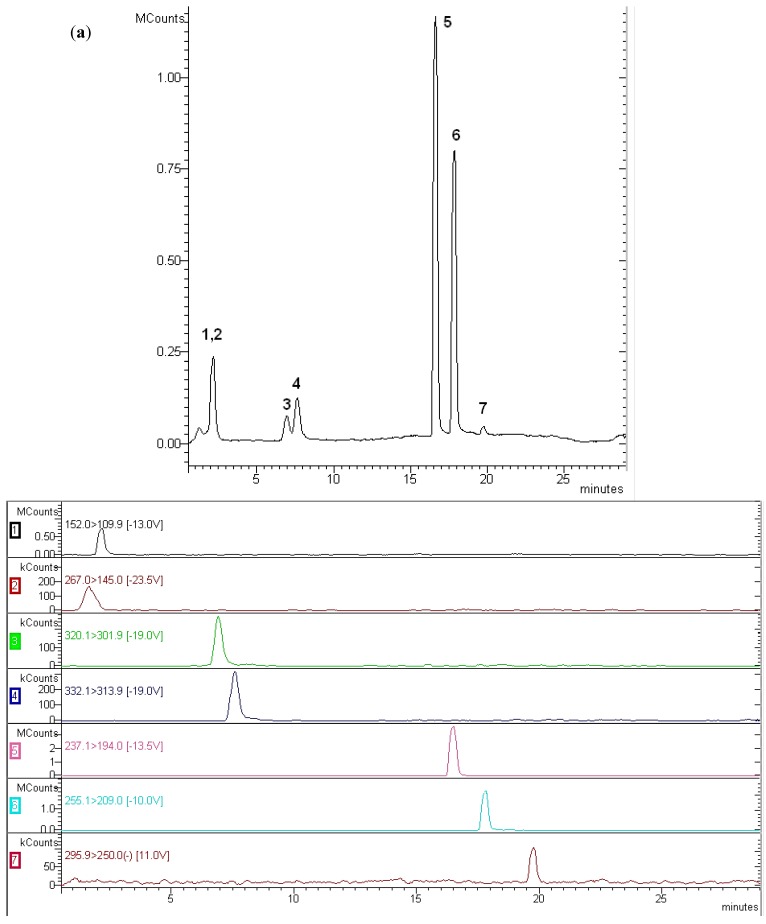
(**a**) Total ion current of pharmaceutical compounds in a standard mixture with LC-MS/MS detection: 1-Atenolol; 2-Acetaminophen; 3-Norfloxacin; 4-Ciprofloxacin; 5-Carbamazepine; 6-Ketoprofen; 7-Diclofenac; (**b**) The fragment ions in the multiple reaction monitoring (MRM) mode.

The optimisation of the mass spectrometer parameters, such as cone voltage and collision gas energy, was carried out by directly injecting standard solutions of each individual compound into the mass spectrometer. The obtained fragment ions and collision potentials are displayed in Table 2. Figure 1 presents the total ion current (TIC) for target analytes (a) and the fragment ions that were achieved in the multiple reaction monitoring (MRM) mode (b). Two transitions were acquired for the confirmation of all analytes.

**Table 2:** Mass spectrometer parameter for the determination of target analytes.

Compound	Precursor ion (*m/z*)	Cone voltage (V)	Fragment ions (collision potential)	Ion mode
Atenolol	267.0	52	145 (23.5) *, 190 (16.5)	ESI +
Acetaminophen	152.0	56	109.9 (13), 92.8 (20)	ESI +
Norfloxacin	320.1	56	301.9 (19), 230.8 (37)	ESI +
Ciprofloxacin	332.1	52	313.9 (19), 230.8 (36)	ESI +
Carbamazepine	237.1	40	194 (13.5), 179.2 (29.5)	ESI +
Ketoprofen	255.1	52	209 (10), 104.9 (18.5)	ESI +
Diclofenac	295.9	32	214.0 (30), 250.0 (11)	ESI −

* Fragment ion used for cuantitation (MRM).

### 2.4. Solid-Phase Extraction

The SPE cartridges used were Oasis HLB (6 mL, 200 mg) from Waters. The cartridges were conditioned with 5 mL of methanol followed by 5 mL of Milli-Q water a flow rate of 10 mL·min^−1^ for each run. Water samples were then loaded onto the cartridges at a flow rate of 5 mL·min^−1^ and thereafter, the cartridges were washed with 5 mL of Milli-Q water at a flow rate of 10 mL·min^−1^ to remove possible interferences. Finally, the cartridges were dried under vacuum for approximately 5 min and further eluted with 2 mL of methanol at 1 mL·min^−1^. Blanks were run to evaluate any carryover during SPE. To perform LC-MS/MS analysis, the eluates were evaporated under a gentle nitrogen stream and reconstituted with 1 mL of LC-MS quality water.

## 3. Results and Discussion

### 3.1. Optimisation of Solid-Phase Extraction

The extraction process was optimised prior to evaluating the presence of pharmaceutical compounds in seawater. The SPE optimisation included the evaluation of the following experimental variables: cartridge type, pH, ionic strength, sample volume and desorption volume. Different cartridges were tested (Oasis HLB, Sep-Pak C_18_, Bond Elut SCX and Lichrolut EN) and it was found that the Oasis HLB cartridge was the best for the majority of compounds. Furthermore, we evaluated different pHs (3, 7 and 8) and ionic strengths (0%, 15% and 30% NaCl) of the sample solution. pH values of 7 and 0% (w/v) NaCl were found to be the optimum values for the extraction. We also tested different sample volumes (100, 250, 500 and 1,000 mL) and found the optimum to be 500 mL. To evaluate the required volume for desorption step, we tested the use of 1 mL and 2 mL of desorption solvent applied in one portion and 2 mL of desorption solvent applied in two 1 mL portions. The best desorption volume for the extraction of the analytes was 2 mL of methanol applied in one portion. In summary, the optimum conditions for the solid phase extraction were to pass 500 mL of sample at pH 7 and 0% (w/v) NaCl through an Oasis HLB cartridge, followed by elution of the analytes with 2 mL of methanol. The eluates were evaporated under a gentle nitrogen stream and reconstituted with 1 mL of LC-MS quality water. These conditions resulted in 500 times more concentrated samples.

### 3.2. Analytical Parameters

The analytical parameters of the method are shown in Table 3. Calibration curves were established for each compound in the range of 0.5–600 µg·L^−1^, and the correlation coefficients were greater than or equal to 0.9906 in all cases. The recoveries of analytes using the optimised method (SPE extraction and LC-MS/MS detection) were evaluated at a concentration of 0.1 µg·L^−1^ for each compound in triplicate. The obtained recoveries were between 78.3% and 98.2%.

**Table 3:** Analytical parameters for SPE-LC-MS/MS method.

	LOD ^a^ (ng·L^−1^)	LOQ ^b^ (ng·L^−1^)	RSD (%)	Recovery (%)	LDR ^c^ (µg·L^−1^)	r^2^
Atenolol	0.1	0.3	6.5	78.3	0.5–600	0.9986
Acetaminophen	0.6	2.0	6.4	95.7	0.5–600	0.9906
Norfloxacin	2.8	9.3	11.8	80.2	0.5–600	0.9964
Ciprofloxacin	1.0	3.4	10.7	81.4	0.5–600	0.9935
Carbamazepine	0.9	2.9	6.0	96.0	0.5–600	0.9926
Ketoprofen	0.1	0.3	1.7	94.8	0.5–600	0.9962
Diclofenac	1.4	4.6	9.8	98.2	0.5–600	0.9936

^a^ Limit of Detection; ^b^ Limit of Quantification; ^c^ Linear Dynamic Range.

Six standard mixtures of pharmaceuticals (0.1 µg·L^−1^ of each compound) were extracted and then injected into the LC-MS/MS to calculate the reproducibility (RSD, %) for each compound. Satisfactory results were achieved for all compounds with RSDs lower than 11.8%. The limit of detection (LOD) was defined as the lowest concentration that gave a signal-to-noise ratio (S/N) equal to 3, and the limit of quantification (LOQ) was defined as the lowest concentration that gave a S/N equal to 10 [19]. LODs and LOQs were in the range of 0.1–2.8 ng·L^−1^ and of 0.3–9.3 ng·L^−1^, respectively. When compared with the results of other authors, we observed that the detection limit of our proposed method was appropriate for the detection of these pharmaceutical compounds [20].

### 3.3. Evaluation of Pharmaceutical Compounds in Seawater

**Figure 2:**
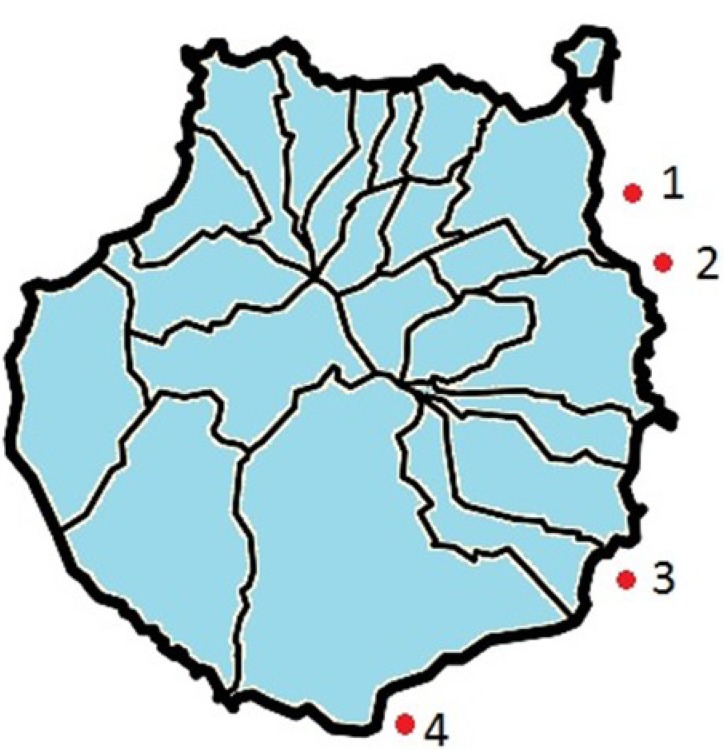
Localisation sampling points on the island of Gran Canaria. 1- Las Palmas de Gran Canaria; 2- Jinámar; 3- Punta Salina; 4- Los Cochinos.

SPE extraction combined with LC-MS/MS was applied to monitor seawater near four different outfalls from urban wastewater located on the island of Gran Canaria (Spain). Figure 2 shows the localisation of sample collection points. Samples were collected during the period between January 2011 and January 2012. Sample collection was not performed during two months (February and April 2011) for the outfalls located in the south of the island due to weather problems.

**Figure 3:**
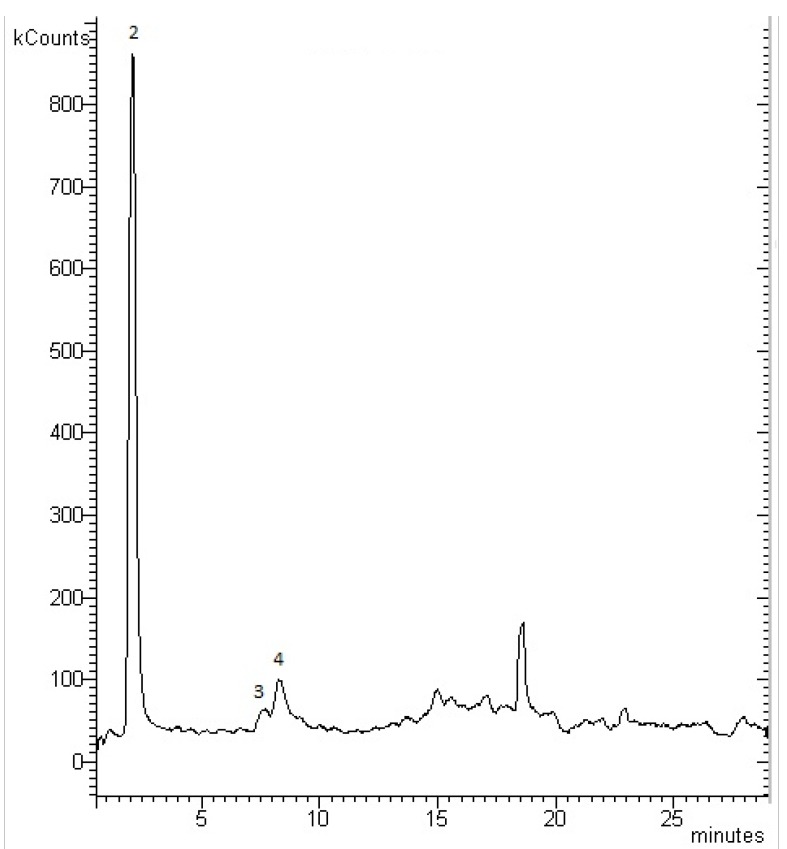
Chromatogram of seawater sample from Las Palmas de Gran Canaria outfall with LC-MS/MS detection: 2-Acetaminophen; 3-Norfloxacin; 4-Ciprofloxacin.

The results of these measurements are shown in Table 4. Forty-eight samples were analysed for the different selected points of the outfalls, and it was observed that two of the analysed pharmaceutical compounds (atenolol and carbamazepine) were not detected in any sample collected. The rest of the compounds under study were found in different concentrations ranging from 4.4 to 3,551.7 ng·L^−1^. Norfloxacin and ciprofloxacin were found in 56.2% and 60.4% of samples, respectively. Low levels of diclofenac, acetaminophen and ketoprofen were found at random. Figure 3 shows a chromatogram corresponding to a sample taken at the Las Palmas de Gran Canaria outfall in March 2011. The presence of acetaminophen, norfloxacin and ciprofloxacin can be observed in this chromatogram.

By analysing the results of each outfall, it can be seen that for the outfalls to the south of the island (Punta Salina and Los Cochinos), higher levels were detected in the second half of the year during the summer and the beginning of winter. This may indicate the existence of higher dump of sewage from different wastewater treatment plants in the south of the island due to an increase in the population because of increased tourism during this time. However, for the outfalls located farther north (Las Palmas de Gran Canaria and Jinámar), the levels were more widely dispersed throughout the year, representing a more stable population in this area. 

The concentrations of pharmaceutical compounds in seawater obtained in this work are similar to those found in other studies for some of the monitored compounds (diclofenac and ketoprofen) [11,21] in seawater. However, we did measure higher levels for some of the monitored compounds, for example, the level of ketoprofen in Jinamar in February 2011.

**Table 4:** Concentrations in ng·L^−1^ found in seawater from four different outfalls in Gran Canaria island. ^a^

Outfall	Date	Acetaminophen	Norfloxacin	Ciprofloxacin	Ketoprofen	Diclofenac
**Las Palmas de Gran Canaria**	Jan-2011	nd ^b^	nd	nd	nd	nd
	Feb-2011	nd	3551.7 ± 315.0	303.6 ± 8.6	67.8 ± 5.3	nd
	Mar-2011	297.0 ± 26.8	1653.0 ± 141.2	123.3 ± 5.4	nd	nd
	Apr-2011	nd	77.9 ± 0.5	nd	nd	nd
	May-2011	nd	nd	nd	nd	nd
	Jun-2011	nd	1248.8 ± 62.3	95.3 ± 0.4	nd	nd
	Jul-2011	nd	nd	40.1 ± 4.9	nd	nd
	Aug-2011	nd	325.5 ± 44.9	18.9 ± 0.2	nd	nd
	Sep-2011	nd	nd	29.64 ± 3.1	nd	47.9 ± 3.5
	Oct-2011	nd	1380.74 ± 197.2	65.9 ± 9.2	nd	28.4 ± 4.0
	Nov-2011	29.7 ± 1.4	69.4 ± 10.3	60.4 ± 7.9	nd	nd
	Dic-2011	nd	nd	nd	41.6 ± 5.0	nd
	Jan-2012	21.5 ± 2.0	11.3 ± 0.7	nd	49.0 ± 3.8	nd
**Jinámar**	Jan-2011	nd	nd	17.4 ± 1.6	nd	nd
	Feb-2011	nd	3179.1 ± 106.2	303.4 ± 12.0	106.3 ± 6.4	nd
	Mar-2011	nd	1130.1 ± 106.6	140.7 ± 21.1	nd	nd
	Apr-2011	nd	92.1 ± 6.0	nd	nd	nd
	May-2011	nd	nd	nd	nd	nd
	Jun-2011	nd	808.6 ± 71.9	66.7 ± 2.0	nd	nd
	Jul-2011	nd	191.6 ± 7.1	70.2 ± 7.9	nd	nd
	Aug-2011	nd	nd	9.0 ± 0.2	nd	nd
	Sep-2011	nd	591.3 ± 15.8	52.3 ± 0.2	nd	nd
	Oct-2011	nd	1565.9 ± 210.0	121.6 ± 15.8	nd	28.4 ± 2.9
	Nov-2011	nd	2250 ± 112.5	119.9 ± 14.9	nd	nd
	Dic-2011	nd	nd	nd	nd	nd
	Jan-2012	nd	18.0 ± 1.0	17.7 ± 1.4	nd	nd
**Punta Salina**	Jan-2011	nd	nd	nd	nd	nd
	Feb-2011	-	-	-	-	-
	Mar-2011	nd	nd	nd	nd	nd
	Apr-2011	-	-	-	-	-
	May-2011	nd	nd	nd	nd	nd
	Jun-2011	nd	nd	nd	nd	nd
	Jul-2011	nd	185.3 ± 22.8	18.8 ± 2.6	nd	nd
	Aug-2011	nd	nd	nd	nd	56.7 ± 5.3
	Sep-2011	nd	634.5 ± 52.0	64.9 ± 10.3	nd	343.6 ± 51.1
	Oct-2011	nd	1681.5 ± 244.0	101.0 ± 5.7	nd	29.5 ± 1.4
	Nov-2011	nd	33.3 ± 2.4	30.8 ± 3.9	nd	nd
	Dic-2011	nd	nd	nd	nd	nd
	Jan-2012	nd	22.9 ± 2.1	26.3 ± 3.8	nd	nd
**Los Cochinos**	Jan-2011	nd	nd	nd	nd	nd
	Feb-2011	-	-	-	-	-
	Mar-2011	nd	nd	nd	nd	nd
	Apr-2011	-	-	-	-	-
	May-2011	nd	nd	nd	nd	nd
	Jun-2011	nd	nd	nd	nd	nd
	Jul-2011	nd	134.3 ± 15.1	80.1 ± 12.2	nd	nd
	Aug-2011	nd	nd	nd	nd	57.1 ± 0.8
	Sep-2011	nd	899.9 ± 130.8	72.0 ± 8.8	nd	160.0 ± 24.4
	Oct-2011	nd	nd	4.4 ± 0.5	nd	23.7 ± 0.4
	Nov-2011	nd	20.8 ± 3.2	15.8 ± 1.4	nd	nd
	Dic-2011	nd	17.4 ± 1.9	12.5 ± 0.2	nd	nd
	Jan-2012	nd	17.0 ± 1.3	13.5 ± 0.1	nd	nd

^a^ n = 3; ^b^ nd = no detected.

Furthermore, the fluoroquinolone levels (norfloxacin and ciprofloxacin) we found were quite high. This fact can be seen by comparing our results with data obtained for the levels of these compounds in the effluents of wastewater treatment plants on the island of Gran Canaria, which did not exceed 13.62 µg·L^−1^ [22]. Upon reaching the seawater, there should be a dilution effect such that the concentrations in seawater are lower than those found in effluent water samples, as is the case in the sampling of norfloxacin in February 2011 at Las Palmas de Gran Canaria (3.6 µg·L^−1^). Fluoroquinolone levels that other authors have found in municipal wastewater effluents and in the receiving surface water [23,24] are lower (309.2 ng·L^−1^ maximum) than levels we found in this study, but this comparison is between different matrices. If we do the comparison with a similar matrix (seawater), the levels found in our study would be therein values obtained in others studies (7.5–103 ng·L^−1^) [25], (3.2–6,800 ng·L^−1^) [26]. 

Although these compounds are expected to be preferentially adsorbed on solid environmental matrices [27], the concentrations found in some cases in seawater are quite high. So, this may be due to the type of antibiotics (ciprofloxacin and norfloxacin) are used in aquaculture, Zou *et al*. suggest that fluoroquinolones (norfloxacin and ciprofloxacin) are extensively used in aquaculture and it is the cause of the high concentrations found for these compounds [26]. The aquaculture is an economic activity of production in Gran Canaria Island that has grown in the last years and it is possible that the unusual concentrations may result from the use of these compounds in fish ponds, which exist close to the sample collection points.

## 4. Conclusions

In the present work, a study of the presence of seven pharmaceutical compounds (atenolol, acetaminophen, norfloxacin, ciprofloxacin, carbamazepine, ketoprofen and diclofenac) in seawater samples near four outfalls from urban wastewater on the island of Gran Canaria in Spain was executed. SPE using an Oasis HLB cartridge was used for the extraction step. Subsequently, detection was performed using liquid chromatography tandem mass spectrometry (LC-MS/MS). Seawater samples were collected monthly in the period between January 2011 and January 2012. During the monitoring time, two of the analysed pharmaceutical compounds (atenolol and carbamazepine) were not detected in any of the collected samples. All compounds under study were found in variable concentrations ranging from 4.4–3,551.7 ng·L^−1^. The fluoroquinolone (norfloxacin and ciprofloxacin) compounds were detected more often and at the highest concentrations in the analysed samples.
